# Predictors of repeated acute hospital attendance for asthma in children: A systematic review and meta‐analysis

**DOI:** 10.1002/ppul.24068

**Published:** 2018-06-05

**Authors:** Cristina Ardura‐Garcia, Marie Stolbrink, Seher Zaidi, Philip J. Cooper, John D. Blakey

**Affiliations:** ^1^ Liverpool School of Tropical Medicine Liverpool UK; ^2^ Facultad de Ciencias Medicas, de la Salud y la Vida Universidad Internacional del Ecuador Quito Ecuador; ^3^ Institute of Infection and Immunity St George's University of London London UK; ^4^ Respiratory Medicine Royal Liverpool Hospital Liverpool UK; ^5^ Health Services Research, Institute of Psychology Health and Society University of Liverpool Liverpool UK

**Keywords:** asthma attacks, emergency department, future risk, hospital admission, paediatric asthma

## Abstract

**Background:**

Asthma attacks are common and have significant physical, psychological, and financial consequences. Improving the assessment of a child's risk of subsequent asthma attacks could support front‐line clinicians’ decisions on augmenting chronic treatment or specialist referral. We aimed to identify predictors for emergency department (ED) or hospital readmission for asthma from the published literature.

**Methods:**

We searched MEDLINE, EMBASE, AMED, PsycINFO, and CINAHL with no language, location, or time restrictions. We retrieved observational studies and randomized controlled trials (RCT) assessing factors (personal and family history, and biomarkers) associated with the risk of ED re‐attendance or hospital readmission for acute childhood asthma.

**Results:**

Three RCTs and 33 observational studies were included, 31 from Anglophone countries and none from Asia or Africa. There was an unclear or high risk of bias in 14 of the studies, including 2 of the RCTs. Previous history of emergency or hospital admissions for asthma, younger age, African‐American ethnicity, and low socioeconomic status increased risk of subsequent ED and hospital readmissions for acute asthma. Female sex and concomitant allergic diseases also predicted hospital readmission.

**Conclusion:**

Despite the global importance of this issue, there are relatively few high quality studies or studies from outside North America. Factors other than symptoms are associated with the risk of emergency re‐attendance for acute asthma among children. Further research is required to better quantify the risk of future attacks and to assess the role of commonly used biomarkers.

## 1 INTRODUCTION

Asthma is the most frequent chronic disease in children^1^ and its worldwide prevalence is still rising, with an estimated 300 million people affected.^2^ For many years, daily symptoms have been the focus of treatment guidelines, and physicians have used control questionnaires to guide treatment adjustments. However, current guidelines^3^, ^4^ have an increasing focus on the risk of adverse outcomes, such as asthma attacks, as this risk is not directly correlated with daily control. Children with largely well‐controlled symptoms are still at risk of developing severe asthma attacks.^5^

Asthma attacks are common^6^ and are associated with high healthcare costs^7^ as well as missed school and workdays. They cause anxiety^8^ and carry a risk of death and long‐term effects such as loss of lung function.^9^ These acute events are especially relevant for children among whom there is the greatest potential for loss of lung function. Attacks often follow a viral respiratory tract infection^10^, ^11^ but secondary care attendance is generally preventable. Attacks may occur despite the use of inhaled corticosteroids,^5^, ^12^ though it is neither affordable nor safe to provide all children with more aggressive treatment, particularly in low‐middle income countries. It is therefore important to be able to identify patients at greatest risk of further attacks and hospital admission to better prioritize limited resources and provide additional education and support or adjustments to treatment where most needed.

Currently, it is not possible to predict which children among those treated for an acute asthma attack, are at a greater risk of suffering repeated attacks. Physicians treating asthmatic patients are identifying rather poorly who is at risk of asthma attacks.^13^ The development of a tool to enable clinicians to identify such children could be useful to optimize treatment strategies and address modifiable risk factors. This is especially relevant when treating patients with discordant manifestations of asthma such as few daily symptoms but evidence from biomarkers of active eosinophilic inflammation in the airways and therefore a high risk of exacerbations, and vice versa,^14^ and when healthcare resources are stretched. Individualizing therapy has the potential to reduce the patient's risk of adverse outcomes from their disease and from medications.

Several factors have been associated with a higher risk of attack among asthmatic children,^15^ both aspects of clinical history such as past attacks and objective measures such as low FEV1. However, findings from large database or cohort studies do not necessarily reflect the population seen in the emergency department (ED) or hospital ward. They therefore may not be informative for the common clinical scenario of reviewing a child in the ED or ward and deciding who needs changes in treatment or specialist referral. We therefore set out to identify predictors (personal and family history, and biomarkers) for subsequent asthma attacks in children attending hospital with an acute episode from the published literature. The aim of the study was to collate information that could support targeted secondary prevention interventions.

## 2 MATERIALS AND METHODS

### 2.1 Data sources and search strategy

We conducted systematic searches of bibliographic databases as described in the Supporting Information (E‐Table S1). All databases were searched from their inception to the present with no language of publication restriction. Searches were carried out by a Cochrane Information Specialist up to 9th January 2017. Duplicate references were removed using reference management software (EndNote X7). The reference list of each selected publication was hand‐searched for relevant studies.

### 2.2 Study selection

Studies with the following criteria were included: (1) cohort and case‐cohort observational design analyzing factors related to asthma clinical history, previous treatment, lung function, biomarkers, or readily measured environmental exposures; or controlled trials that involve a lifestyle or social (not educational or pharmaceutical) interventions; (2) asthmatic children aged between 5 and 15 years old (among the age range of the study) recruited from the ED or ward, treated for an acute asthma exacerbation, included as participants; (3) emergency re‐attendance or hospital readmission due to an asthma attack listed as outcomes.

The list of abstract and titles was reviewed to exclude publications that were clearly not contributory on this basis and duplicate titles. Full text articles of selected papers were obtained via University library and inter‐library loan and reviewed for eligibility, excluding those not fulfilling inclusion criteria.

### 2.3 Data extraction

Data were extracted by three independent authors using a standard data extraction form. Possible disagreements were resolved by discussion. RevMan 5 and Endnote X7 software were used to assist in the collection and management of data from abstracts and papers.

### 2.4 Quality and risk of bias assessment

Studies’ accuracy and risk of bias was assessed using the criteria of the Cochrane Handbook for Systematic Reviews of Interventions by three independent authors. Observational studies’ quality was also assessed using the Newcastle‐Ottawa Quality Assessment Scale,^16^ with a score of seven or more defined as of high methodological quality.

### 2.5 Analysis

Data from comparable studies were combined in quantitative analyses. We pooled data using a random effect model in RevMan5, creating pooled estimates of effects for Hazard and Odds Ratios separately. Reports that presented the results using mean values, with no other data, were not included in the meta‐analyses. We used the generic inverse variance as the analysis method for some of the pooled estimate of effects, as some studies only reported Odds or Hazard Ratios.

We represented an estimate of the degree of variation between study outcomes using the *I* ^2^ statistic.^17^ Overall pooled estimates of effect are presented on the basis of being informative to some degree if *I* ^2^ was high, if either there were few studies (a situation where *I* ^2^ can be imprecise or biased), or if the studies found a consistent direction of effect which would imply that factor was worth consideration in future prospective studies.

## 3 RESULTS

**Figure 1:**
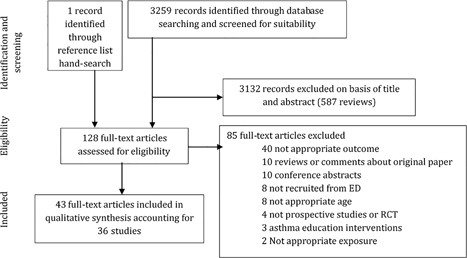
Flow diagram of included and excluded studies

A total of 3259 records were identified and screened for eligibility with one additional paper obtained through reference list screening (Figure 1). Forty‐three papers fulfilled our inclusion criteria after full‐text screening, accounting for 36 studies.

### 3.1 Studies’ characteristics and definitions

#### 3.1.1 Design, participants, and setting

Twenty‐three studies were retrospective analyses of health‐care databases. There were six prospective cohorts, three randomized‐controlled trials (RCT), and four with other prospective designs. Most studies were carried out in North America (21 in USA and 3 in Canada). Participants were recruited among inpatients admitted for acute asthma in most of the studies (27) (Table 1).

**Table 1:** Studies’ characteristics

Study design	Study	Location	Sample selection and setting	Age (years)	Sample size	Years	Recruit‐ment	Outcomes measured
RCT	Gorelick^39^	US (Milwauke)	Simple random allocation. Hospital based. Intervention: improve linkage of patients back to primary care. FU: 6 months. LTFU: 22%	2‐18	352	2003‐2004	ED	ED within 6 months
	Kercsmar^40^	US (Ohio)	Stratified random permuted block scheme. Hospital, primary care and other community sources. Intervention: home remediation of moisture sources. FU: 12 m. LTFU: 10% intervention, 24% control	2‐17	62	ND	≥2 ED or hosp. admis. last year	ED or hospital admission
	Madge^41^	UK (Glasgow)	Simple random allocation. Hospital based. Intervention: educational and training programme. FU: 2–14 months. LTFU: 0%	2‐14	201	1994‐1995	Inpatient	ED and hospital admission
Cohort	Bloomberg^42^	US (St Louis)	Review of hospital database All eligible. Hospital‐based study. FU: up to 1 year. LTFU: 0%	0‐20	8761	1999	Inpatient	Single versus multiple admissions
	Brittan^43^	US (Colorado)	Review of health database. All eligible. Hospital‐based. FU: 15–90 days LTFU: 0%	2‐18	9288	2009‐2011	Inpatient	Hospital admission
	Camargo^44^	US (Florida)	Medicaid claims review. All eligible. Hospital‐based. Follow‐up: 12 months	0‐8	10 976	1999‐2001	ED/Inpatient	ED or hospital admission
	Chabra^45^	US (California)	Hospital discharge data review. All eligible. Non‐federal acute care hospitals. Only Medicaid patients.	1‐12	4947	1994	Inpatient	Single vs multiple hospital admission
	Chen^46^	US (St Louis)	Prospective. All eligible. Hospital‐based study. FU: 1 year. LTFU: 0%	4‐18	115	1999	Inpatient	Hospital admission
	Chen^47^	Canada (Ottawa)	Review of national hospital discharge data All eligible. Hospital‐based study. FU: 1m − 3 years. LTFU: 0%	0‐20	60 641/86 863*	1994‐1997	Inpatient	Hospital admission
	Cincinnati^22^, ^48^, ^49^, ^50^	US (Cincinnati)	Prospective. All eligible. Hospital‐based study. FU 12–14 months. LTFU: 0%	1‐16	601	2008‐2009	Inpatient	ED or hospital admission
	GCARS^35^, ^51^, ^52^, ^53^, ^54^	US (Cincinnati)	Prospective. All eligible. Hospital‐based study. FU 12 months. LTFU: 0%	1‐16	774	2010‐2011	Inpatient	Hospital admission
	Giarola^55^	Australia (Darwin)	Review of electronic hospital database. All eligible. Hospital‐based study. FU 12 months. LTFU: 0%	≤ 15	200	2005/6 2010/1	Inpatient	Hospital admission
	Gurkan^56^	Turkey	Review of hospital admissions. All eligible. Hospital‐based study. FU: 13–48 months. LTFU: 0%	3‐15	140	1995‐1997	Inpatient	Hospital admission
	Kenyon^57^	US	Review of health database. All eligible. Hospital‐based study. FU: 12 months. LTFU: 0%	≥ 2	36 601	2008‐2010	Inpatient	Hospital admission at 15,30,60,80,365 d
Cohort	Kenyon^58^	US	Review of health database. All eligible. Hospital‐based study. FU: 3 months. LTFU: 0%	2‐18	31 658	2006‐2007	Inpatient	Hospital admission
	Kocevar^59^	Norway	Review of national inpatient database. All eligible. Hospital‐based study. FU: up to 2 years. LTFU: 0%	0‐14	2961	1998‐1999	Inpatient	Hospital admission
	Lasmar^60^	Brazil (Belo Horizonte)	Review of hospital admissions. All eligible. Hospital‐based study. FU: Maximum 24 months. LTFU: 0%	< 15	202	1994‐1995	Inpatient	Hospital admission
	Li^61^	Canada (Ontario)	Multiple linked health administrative datasets review. All eligible. Hospital based. Follow‐up: 12 months. LTFU: 0%	2‐17	29391	2006‐2009	ED	ED and hospital admission
	Liu^18^	US (Rhode Island)	Review of hospital admissions. All eligible. Hospital‐based study. FU: 5 years. LTFU: 0%	0‐18	2913	2001‐2005	Inpatient	Hospital admission
	Minkovitz^62^	US ()	Review of hospital admissions. All eligible. Hospital‐based study. FU: 12 months. LTFU: 0%	0‐14	119	1994‐1995	Inpatient	Hospital admission
	Mitchell^63^	New Zealand (Auckland)	Review of hospital admissions. All eligible. Hospital‐based study. FU: Maximum 33 months. LTFU: 0%	0‐14	1034	1986‐1987	Inpatient	Hospital admission
	Morse^64^	US	Multicentre review of databases. Simple random. Hospital based. Follow‐up: 3 months‐3 years. RR: 60% of all freestanding children's hospitals.	2‐17	37267	2008‐2010	Inpatient	ED attendance at 30 and 90 days
	Rodriguez‐Martinez^65^	Colombia	Prospective. Convenience sample. Hospital‐based study. FU: 12 months. LTFU: 0%	1‐18	101	?	Inpatient	Hospital admission
	Rasmussen^66^	New Zealand	Prospective. All eligible. Included only those admitted to hospital for asthma. Hospital‐based study. FU: 26 years. LTFU: 18 died (from total cohort)	0	62 (766 wheeze)	Born 1972‐3	Birth cohort	Single vs multiple hospital admission
	Rushw^67^	Australia (New South Wales)	Review of Inpatient Statistics Collection. All eligible. Hospital‐based study. FU: Maximum 6 months. LTFU: 0%	1‐14 (1–44)	(15682)	1989‐1990	Inpatient	Hospital admission
	Smiley^68^	US	Review of Department of Defence Military Health System database. All eligible. Hospital based. FU: 12 months LTFU: 0% (15% incomplete data)	2‐17	10460	2010‐2013	ED	ED
	Sporik^69^	UK	Prospective. All eligible. Hospital based. FU: 6 months. LTFU: 11%	1‐15	82	1988‐1989	Inpatient	Hospital admission within 6 months
	Taylor^20^	Canada (Nova Scotia)	Review of ED billing and admission databases. All eligible. Hospital based. FU: 6 months. LTFU: 0%	Children (< 18)	16994	1997	ED	ED and hospital admission
Cohort	Tolomeo^70^	US (New England)	Review of medical records. Convenience sample. Hospital based. FU: 12 months. LTFU: 0%	2‐15	298	2006	Inpatient	ED and hospital admission
	Wallace^19^	US (New Jersey)	Review of hospital admissions. All eligible. Hospital‐based study. FU: 180 days. LTFU: 9% (incomplete data)	1‐14	21 016	1994‐2000	Inpatient	Hospital admission
	Wu^71^	US	Retrospective cohort study of participants in RCT. Hospital‐based. All eligible. FU: 12 months. LTFU: 0%	1‐17	108	?	ED	ED and hospital admission
	Zipkin^21^	US (Los Angeles)	Retrospective cohort study. Hospital‐based. All eligible. FU: 12 months. LTFU: 0%	2‐17	1176	2006‐2013	Inpatient	ED or hospital admission
Other	Bergert^72^	Hawaii	Before and after quality improvement study. Hospital‐based intervention. No sample selection. LTFU: 0%	2‐18	763	2006‐2012	ED	ED or hospital admission
	Davis^73^	US (California)	Matched cohort with non‐randomly applied intervention. Intervention for all eligible. Controls: Matched by age and past hospital care utilization. Hospital‐based. FU: 1 year LTFU: 0%	1‐18	1396	2005‐2007	Inpatient	ED
	Fassl^74^	US (Salt Lake City)	Before and after quality improvement study. Hospital‐based intervention. No sample selection. LTFU: 0%	2‐17	1865	2005‐2011	Inpatient	ED or hospital admis. within 6 m
	Vicendese^75^	Australia (Melbourne)	Case‐control, nested in cohort study. Cases at least 2 admissions, controls only 1 admission. Non‐random. Hospital based.	2‐17	44 (22 cases)	2009‐2011	Inpatient	Hospital admission

GCASR, Greater Cincinnati Asthma Rik Study; ND, not determined; LTFU, lost to follow‐up; RR, response rate; ED, emergency department; dx, diagnosis; y, years; m months.

#### 3.1.2 Outcome

The study outcome was ED re‐attendance in 4 reports, ED or hospital readmission in 6, hospital readmission alone in 21 studies and a further 5 studies analyzed data for both outcomes separately.

#### 3.1.3 Predictors

The risk factors or predictors studied varied amongst studies, including: socio‐demographic characteristics such as age, gender, sex, and socioeconomic status (SES, including household/neighborhood income, private vs public insurance and working rank); and asthma characteristics (severity, treatment, previous admissions).

### 3.2 Risk of bias

The number of reports with low, unclear, or high risk of bias was 22, 7, and 7, respectively. Those with an unclear risk of bias lacked information on relevant aspects of the methods, mainly sample selection, or did not state clearly the number or factors studied in order to assess reporting bias. The details of the risk of bias assessments are shown in E‐Table S2.

### 3.3 Predictors

#### 3.3.1 Factors related to the person

##### Age

The effect of age on the future risk of ED or hospital readmission was examined in 16 studies (E‐Table S3). There was a marked variation in the age group classification and statistical methods used for the comparisons, precluding a meta‐analysis of this factor. Studies were consistent in reporting that younger children had a higher risk of ED or hospital readmission.

##### Sex

**Figure 2:**
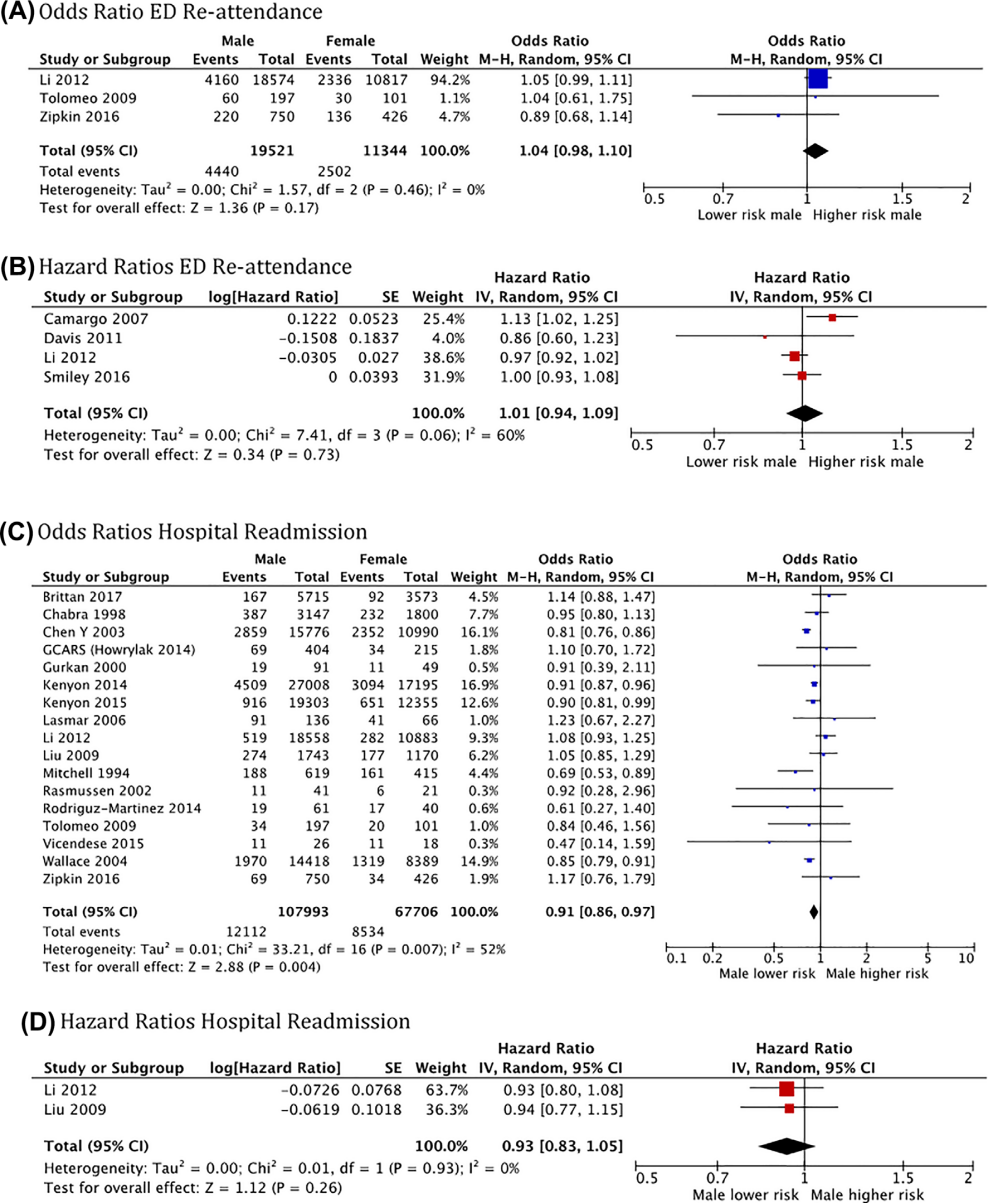
Forest plots for the association of sex with emergency department re‐attendance and hospital readmission for acute asthma in children using a random effects model. (Figure 2A‐2D showing separate estimations for odds and hazard ratios)

Six reports analyzed sex as a risk factor for ED and 17 for hospital readmission, though some of them stratified its effect by age. There was a decreased odds of hospital readmission among boys compared to girls (OR 0.91, 95%CI: 0.86‐0.97; *N* = 67706; *I* ^2^ = 52%) in the pooled analysis of data from 17 studies (Figure 2C), but no difference in the pooled analysis of other two studies reporting hazard ratios (Figure 2D). There was no difference in ED re‐attendance by sex in the studies reporting either odds or hazard ratios (Figures 2A and 2B).

##### Ethnicity

**Figure 3:**
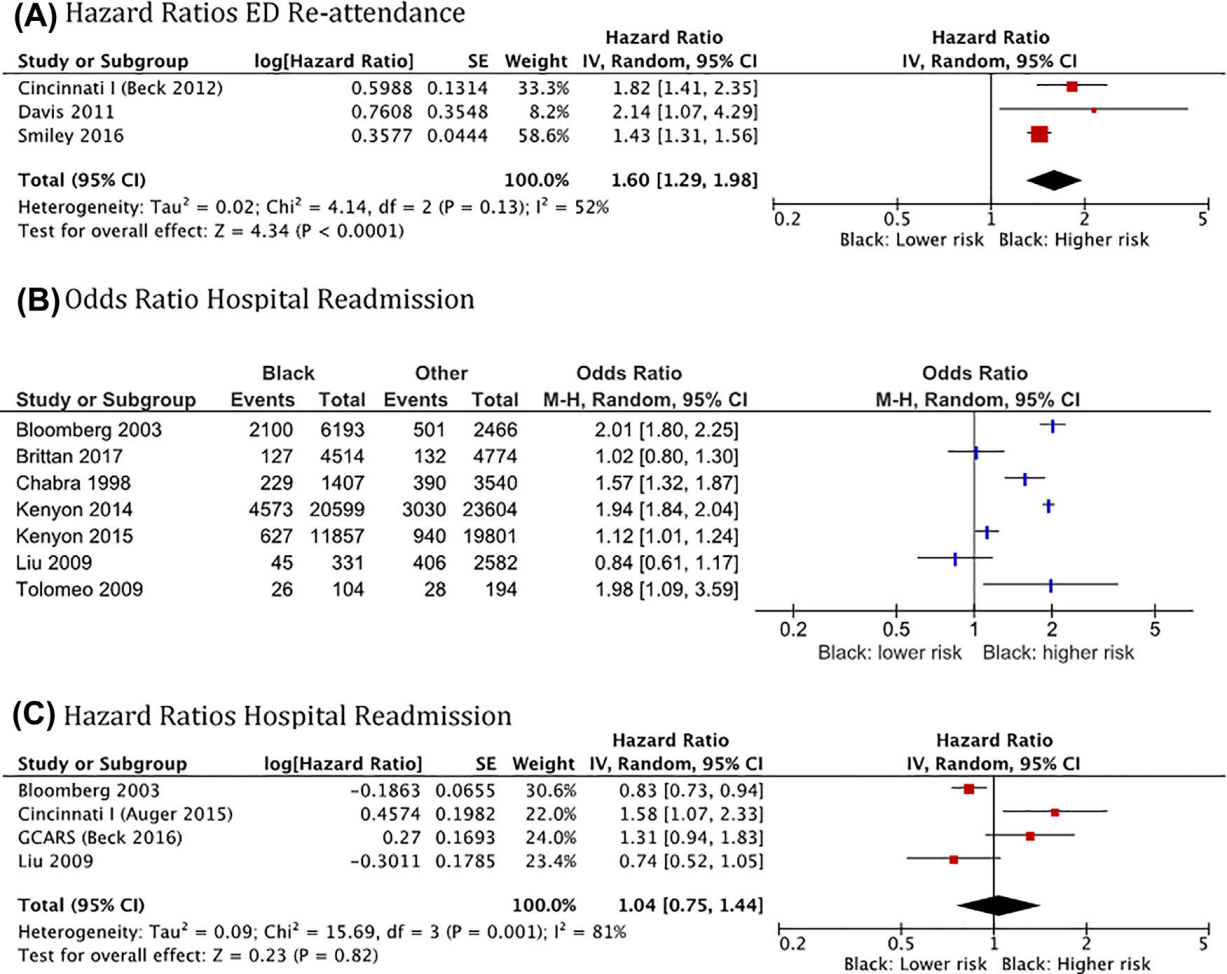
Forest plots for the associations of ethnicity (black vs other) with emergency department re‐attendance and hospital readmission for acute asthma in children using a random effects model

The effect of ethnicity on ED or hospital readmission was included in 18 reports, using different classifications. The most frequent was the comparison between black or African‐American and white or other origins, and the risk of readmission for asthma. There was an increased rate of ED re‐attendance among African‐American compared to white children (HR: 1.60, 95%CI: 1.29–1.98; *N* = 12457; *I* ^2^: 52%) in the pooled analysis of three studies reporting Hazard Ratios (Figure 3A). The results of seven studies analyzing the association between black ethnicity and the odds of hospital readmission for acute asthma are shown in Figure 3B. The pooled result is not shown given the marked heterogeneity in study results (*I* ^2^ = 95%). Four other studies reporting Hazard Ratios for hospital readmission showed no association between black ethnicity and hospital readmission rate (Figure 3C), but again were apparently heterogeneous in their findings (*I* ^2^ = 81%). One paper studying hospital readmission was excluded from the meta‐analysis as results were stratified by age and sex.^19^

##### Socioeconomic status (SES)

**Figure 4:**
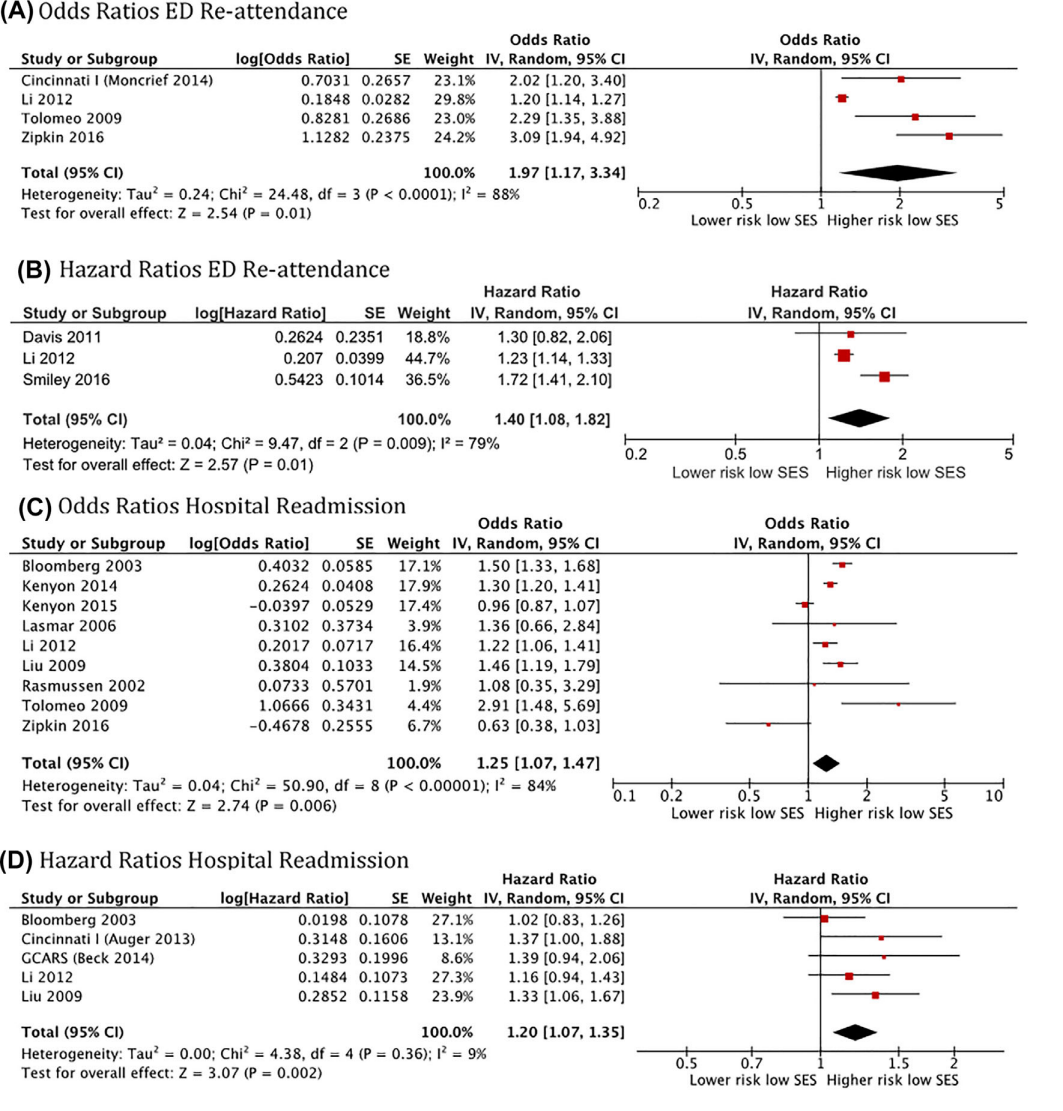
Forest plots for the associations of socioeconomic status (SES) with emergency department re‐attendance and hospital readmission for acute asthma in children using a random effects model

Twenty‐one reports examined SES as a predictor for ED or hospital readmission for asthma. The specific predictor used differed, with public insurance (vs private or other) or low household income being the most frequent markers adopted for low SES. A meta‐analysis of data from four studies showed increased odds of ED re‐attendance for acute asthma in children of low SES (OR: 1.23; 1.17‐1.30; *N* = 31466) (Figure 4A), consistent with the pooled analysis of other three studies reporting HR (HR: 1.40, 95%CI: 1.08‐1.82; *N* = 41,247) (Figure 4B). Both analyses show a consistent direction of effect but there was significant heterogeneity (*I* ^2^: 88% and 79%, respectively), consistent with the variation in study design and predictor used.

A further nine studies reporting odds ratios and five studies reporting Hazard ratios were used to analyze the effect of low SES on hospital readmission for acute asthma, producing a pooled OR: 1.25 (95%CI: 1.07‐1.47; *N* = 111062; I^2^: 84%) (Figure 4C) and a pooled HR: 1.20 (95%CI: 1.07‐1.35; *N* = 42440; *I* ^2^: 9%)) (Figure 4D).

##### Comorbidities

Seven reports analyzed the risk of hospital readmission among children with other concomitant allergic diseases, including allergic conjunctivitis, allergic rhinitis, or eczema. The pooled OR of six of these studies showed an increased risk of hospital readmission for asthma for children with concomitant allergic diseases (OR: 1.90, 95%CI: 1.43‐2.52; *N* = 32387; *I* ^2^: 44%) (E‐Figure S1).

#### 3.3.2 Factors related to asthma characteristics

##### Previous ED or hospital admission

**Figure 5:**
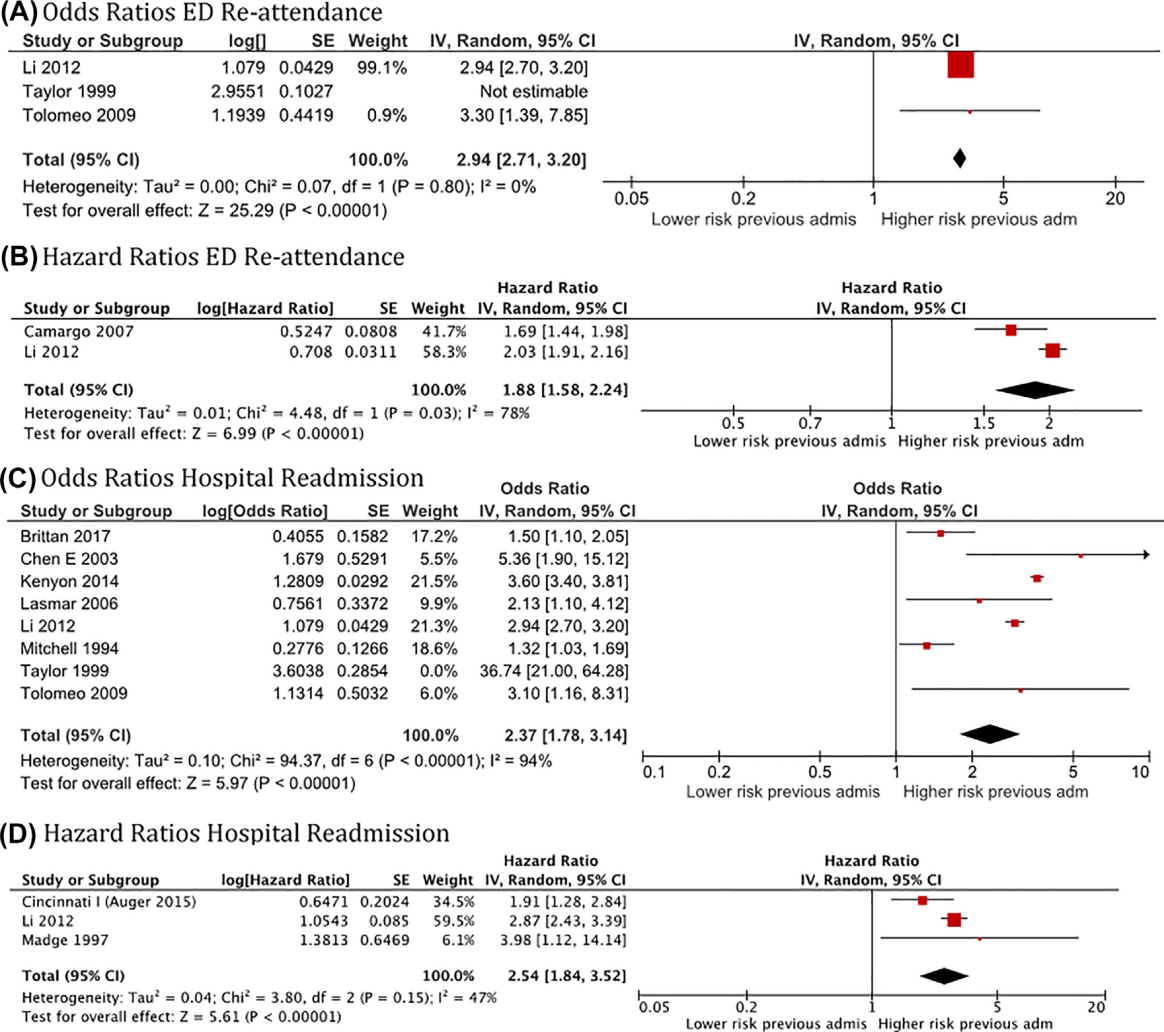
Forest plots for the associations of previous ED or hospital admissions for acute asthma with emergency department re‐attendance and hospital readmission for acute asthma in children using a random effects model

Eight reports included data on the risk of ED or hospital readmission according to a history of previous hospital or ED admissions, either in the previous 12‐24 months (the most common) or ever in life. The three reports studying ED re‐attendance odds ratios are shown in Figure 5A. The pooled OR for two of these studies was 2.94 (95%CI: 2.71‐3.20; *N* = 29689; *I* ^2:^ 0%). The results from one of the studies (Taylor 1999) was not included in the meta‐analysis and forest plot as it pooled together all children who had not had an asthma attack in the baseline year (whether they had asthma or not).

The two reports studying hazard ratios (HR: 1.88, 95%CI: 1.58‐2.24; *N* = 40367; *I* ^2^: 78%), showed an increased rate among children with a history of previous ED or hospital admissions for acute asthma (Figure 5B). The same occurred with the eight studies reporting odds ratio (OR: 2.37, 95%CI: 1.78‐3.14; *N* = 76929; *I* ^2^: 94%) and the three studies reporting hazard ratios for hospital readmission (HR: 2.54, 95%CI: 1.84‐3.52; *N* = 30193; *I* ^2^: 47%) (Figures 5C and 5D). One of the studies (Taylor 1999) was not included in the pooled analysis of the odds ratio for hospital readmissions, for the same reason as exposed above. Although there was apparent heterogeneity between studies in these analyses, the direction of effect was consistent and individual studies found similar effect sizes so indicative pooled effect sizes are shown.

##### Asthma severity and controller treatment

Ten studies assessed the association between asthma severity, control or controller treatment received with the risk of ED or hospital readmission (E‐Table S4). The type of predictor and definition of severity varied greatly between studies, precluding a meta‐analysis. The findings related to severity as defined by treatment were inconsistent.

#### 3.3.3 Factors related to potential follow‐up

##### Asthma follow‐up

Ten papers examined aspects of follow‐up after the index ED or hospital admission (E‐Table S5). Three studies explored the effect of the Children's Asthma Care (CAC) measures set implementation, which comprises providing reliever medication and systemic corticosteroids for children admitted to hospital for asthma, and discharging them with a home management plan. They showed no effect on ED re‐attendance after the initial admission for asthma and two of them demonstrated a reduced risk of hospital re‐admission after the implementation of the CAC measures. One further study showed a decreased risk of both hospital readmission and ED re‐utilization for acute asthma after Home Management Plan Care implementation (part of CAC).^21^

Five studies analyzed the effect of different follow‐up visit characteristics and ED or hospital readmission for asthma with disparate outcomes. Three reports studied the effect of receiving an asthma action plan at discharge on the risk of ED or hospital readmission. Two of them were combined (E‐Figure S2) showing no association.

#### 3.3.4 Other factors

##### Exposure to tobacco smoke (ETS)

Five studies included data on ETS, producing a pooled odds ratio of 1.60 (95%CI: 0.94‐2.72; *N* = 1041; *I* ^2^: 49%) for hospital readmission for acute asthma for those exposed to tobacco smoke (E‐Figure S3). One other paper^22^ measuring Hazard ratio, showed no association between ETS and hospital readmission rate for asthma (AHR: 0.83, 95%CI: not published, *P‐*value: not significant).

Other factors included: family history of asthma or allergic diseases, parental level of education, personal history (previous immunizations, early life feeding methods), caregiver's situation (psychological stress, beliefs, knowledge, marital status, or concerns), asthma triggers, exposure to allergens or pollution and home characteristics, adherence to asthma treatment, characteristics of index asthma admission, and home remediation interventions of water infiltration. The outcome definitions and specific exposures studied were highly heterogeneous between studies meaning no robust consensus conclusions could be drawn.

## 4 DISCUSSION

### 4.1 Summary of main findings

Asthma is a very common reason for emergency attendance. Clinicians are therefore often faced with decisions on whether to increase treatment and who to refer for a specialist opinion. This systematic review and meta‐analysis has identified a history of previous ED or hospital admissions as the major risk factor for emergency care and hospital readmissions for acute asthma in children. Further, our results indicate that children of African‐American ethnicity (compared to white or other ethnicity), low socioeconomic status (as measured by having public insurance or low family income), with concomitant allergic diseases (allergic rhinitis/rhinoconjunctivitis or eczema) and being younger than 5 years of age, appear at a greater risk of subsequent emergency care visits or hospital readmissions for acute asthma.

### 4.2 Strengths

This systematic review answers a relevant question with public health implications for future asthma management. It has been developed in accordance with best practice and using an extensive search with no time or language publication restrictions to ensure the inclusion of potentially suitable studies. Risk of bias was assessed and presented for each included study separately. We were also able to undertake a quantitative analysis of the most relevant predictors, increasing the relevance of our findings.

### 4.3 Limitations

The data collected had a moderate to low strength of evidence, due to the quality of the included studies and the inconsistent findings in some of the factors reported.^23^, ^24^ Most of observational studies included were retrospective cohorts using hospital or insurance databases with a large number of patients (more than 10 000 participants in six studies). However, unclear and high risk of bias were common because of inadequate sampling and reporting of only significant results in more than 50% of the observational studies. The RCT's included in the review had some important risk of biases which precluded increasing the strength of evidence of the review.

Variation in study design and outcomes precluded meta‐analysis for some factors. In other instances, meta‐analyses demonstrated significant variation between studies when compared to error within studies (high *I* ^2^). In most of these cases we present pooled effect size to give an indication of the scale and direction of effect. However, it is not currently possible to derive precise estimates of effect size based on the available literature, and well‐designed prospective studies are required.

Despite a wide literature search with no language restriction, all selected papers were published in English and the clear majority were developed in Anglophone countries, mainly in the US. This likely reflects the fact that asthma has been an important public health problem in these countries for a long period^2^ but limits the generalizability of the findings to other relevant regions such as Latin America were asthma has emerged as an important public health issue.^25^ Decisions on who should be the focus of treatment are crucial in such settings where resources are likely to be very limited. Another important factor is the inclusion of children younger than 2 years old in several studies, an age at which it is difficult to ascertain an asthma diagnosis.^26^ The outcomes used in the studies are also a possible source of bias, as there is no consensus on when a child should be admitted to the ED or hospital for acute asthma. However, this is the current definition used by the ATS for an episode of severe asthma.^27^

### 4.4 Findings in relation to other studies

The use of different study designs and effect measures meant that for most factors under study there was not a simple way to summarize the effect of a given risk factor. However, the effect sizes reported were similar in the most relevant predictors identified, such as low socio‐economic status or history of ED or hospital admission for acute asthma during the previous year. In particular, we did not find a substantial difference for any given risk factor between effect sizes in studies considering OR or HR: That is to say there was no clear difference in effect size when considering whether an exacerbation would happen in the follow‐up period, and frequency of exacerbations. This is likely to be because most children did not have multiple exacerbations during follow‐up. There are also statistical challenges of understanding factors that influence the time to event for a potentially recurrent event.

Children younger than 5 years old were at a higher risk of ED or hospital readmission for acute asthma when compared to different age groups in more than half of the studies that explored age as a predictor of future risk. Preschool children suffer a larger number of acute asthma attacks driven mostly by respiratory viruses.^28^, ^29^ It is also difficult to diagnose asthma in this age group,^26^ potentially leading to inadequate management. Lintzenich et al^30^ showed that children 1‐6 years old hospitalized for asthma were less likely to receive ICS baseline treatment and asthma education than older children.

Lower SES was associated with a higher risk and a higher rate of ED or hospital readmission.^31^ Similar associations had been previously described for other diseases.^32^ It may reflect poorer long‐term management due to inadequate access to primary and specialist care, and that caregivers may be less able to adequately manage a long‐term condition thus relying more on ED attendance. Flores et al^33^ showed that among ethnic minority children with asthma in urban settings, poorer children were less likely to have an asthma specialist than wealthier children.

Children of African‐American origin living in Anglophone countries were at higher risk of re‐attendance for asthma. Non‐white ethnicity has also been described as a predictor of hospitalization or ED visits in adults with severe or difficult‐to‐treat asthma.^34^ Beck et al^35^ reported that up to 80% of the readmission disparity between African‐American and white asthmatic children could be explained by other associated factors, such as access to care or disease management. However, it is uncertain how applicable these findings are outside an urban United States setting.

Co‐existing allergic diseases (allergic rhinitis/rhinoconjunctivitis and eczema) were also associated with a greater risk of hospital readmission for asthma. Previous work has shown that treatment for allergic rhinitis (nasal corticosteroids and antihistamines) is associated with lower rates of unscheduled care use.^36^ This may indicate that untreated comorbid allergic diseases are associated with higher risk of asthma hospital readmissions.

Asthmatic children with a history of a previous ED or hospital admission for acute asthma had 2‐5.8 times more risk of ED re‐attendance and 2.5‐3 times more risk of a hospital readmission. This was therefore the clearest factor related to asthma that was associated with future risk. Similarly, other studies have identified several variables related to previous healthcare utilization for acute asthma that are associated with future risk of severe asthma attacks.^37^, ^38^

## 5 CONCLUSION

In conclusion, we have identified individual‐level and factors related to asthma severity that are associated with a greater risk of future asthma attacks requiring emergency care or hospital readmission. This description of the current evidence base and its limitations could help inform future prospective studies that robustly assess the magnitude and interaction of such risk factors. In future, being able to identify children at risk of future asthma attacks requiring emergency care will guide specific interventions such as educational sessions, management of comorbidities, and personalized treatment adjustments. This approach has the potential to reduce the chance of long‐term complications such as loss of lung function, psychological morbidity, and death.

## Supporting information

Additional supporting information may be found online in the Supporting Information section at the end of the article.

**Figure S1**. Forest plot for the associations of concomitant allergic diseases (allergic rhinitis /rhinoconjunctivitis or eczema) with hospital readmission for acute asthma in children using a random effects model (odds ratios).
**Figure S2**. Forest plots for the associations of being offered an asthma action plan at discharge with emergency department re‐attendance or hospital readmission for acute asthma in children using a random effects model (odds ratios).
**Figure S3**. Forest plots for the associations of second‐hand tobacco smoke exposure (ETS) with hospital readmission for acute asthma in children using a random effects model (odds ratios).

**Table S1**. Search report Asthma exacerbation risk factors in children searches. October 2013 model (odds ratios).
**Table S2**. Statistical Methods and Risk of Bias
**Table S3**. Association between age and risk of ED or hospital readmissions
**Table S4**. Association between asthma severity, control and baseline treatment and risk of ED or hospital readmissions
**Table S5**. Association between asthma follow‐up and management after index admission or ED visit for asthma and risk of ED or hospital readmissions
