# Supplementary material for: Predictors of repeated acute hospital attendance for asthma in children: A systematic review and meta‐analysis
Source: Pediatr Pulmonol. 2018 Jun 5;53(9):1179–92. doi: 10.1002/ppul.24068 (PMC6175073; doi:10.1002/ppul.24068)

**IMAGE LEGENDS SUPPLEMENTARY FIGURES**

E-Figure 1: Forest plot for the associations of concomitant allergic diseases (allergic rhinitis /rhinoconjunctivitis or eczema) with hospital readmission for acute asthma in children using a random effects model (odds ratios).


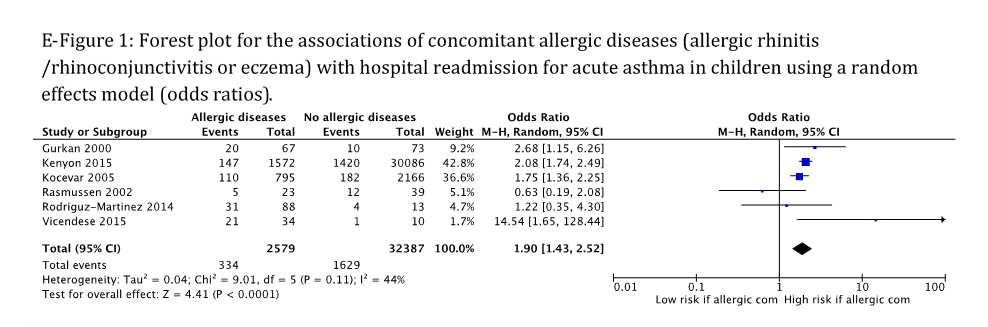


E-Figure 2: Forest plots for the associations of being offered an asthma action plan at discharge with emergency department re-attendance or hospital readmission for acute asthma in children using a random effects model (odds ratios).


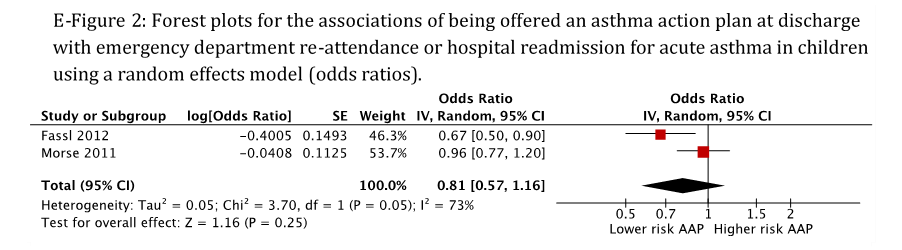


E-Figure 3: Forest plots for the associations of second-hand tobacco smoke exposure (ETS) with hospital readmission for acute asthma in children using a random effects model (odds ratios).


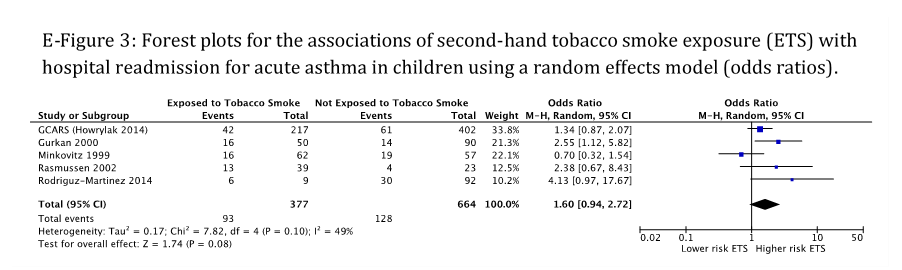

Supplement: Supplementary file 1 — Figure S1. Forest plot for the associations of concomitant allergic diseases (allergic rhinitis /rhinoconjunctivitis or eczema) with hospital readmission for acute asthma in children using a random effects model (odds ratios). Figure S2. Forest plots for the associations of being offered an asthma action plan at discharge with emergency department re‐attendance or hospital readmission for acute asthma in children using a random effects model (odds ratios). Figure S3. Forest plots for the associations of second‐hand tobacco smoke exposure (ETS) with hospital readmission for acute asthma in children using a random effects model (odds ratios). [file PPUL-53-1179-s001.docx]
