# Supplementary material for: Predictors of repeated acute hospital attendance for asthma in children: A systematic review and meta‐analysis
Source: Pediatr Pulmonol. 2018 Jun 5;53(9):1179–92. doi: 10.1002/ppul.24068 (PMC6175073; doi:10.1002/ppul.24068)
Supplement: Supplementary file 2 — Table S1. Search report Asthma exacerbation risk factors in children searches. October 2013 model (odds ratios). Table S2. Statistical Methods and Risk of Bias Table S3. Association between age and risk of ED or hospital readmissions Table S4. Association between asthma severity, control and baseline treatment and risk of ED or hospital readmissions Table S5. Association between asthma follow‐up and management after index admission or ED visit for asthma and risk of ED or hospital readmissions [file PPUL-53-1179-s002.docx]

**SUPPLEMENTARY MATERIAL**

**E-Table 1: Search report Asthma exacerbation risk factors in children searches. October 2013**

| **Search No.** | **Date** | **Database searched** | **Hits (before duplicate removal)** |
| --- | --- | --- | --- |
| 1 | 9/01/2017 | Medline (Pubmed) | 1835 |
| 2 | 9/01/2017 | Cinahl (EBSCOhost) | 353 |
| 4 | 9/01/2017 | PsycInfo (EBSCOhost) | 90 |
| 5 | 9/01/2017 | Embase (OVID) | 2603 |
| 6 | 9/01/2017 | AMED (EBSCOhost) | 3 |
| 7 | 9/01/2017 | WHO clinical trial registry platform (WHO ICTRP) | 8 |
| **FINAL NUMBER OF REFERENCES IN ENDNOTE AFTER DELETING DUPLICATES = 3259** | | | |

**Search strategy**

(risk OR predic* OR associat* OR probabil*) OR "Risk Factors"

AND

(((((asthma) AND hospital admission)) OR ((((asthma AND exacerbation)) OR ((asthma) AND "Disease Progression")) OR (asthmatic AND exacerbation)))) OR (asthma AND "Patient Readmission")

AND

child OR children OR childhood OR pediatr* OR paediatr*

**E-Table 2: Statistical Methods and Risk of Bias**

| Study Design | Study | Analysis Method | Adjust-ment^a^ | Multiple testing | Risk of Bias | Source risk of bias | N-O Score |
| --- | --- | --- | --- | --- | --- | --- | --- |
| RCT | Gorelick 2006 | Analysis per risk factor | NA | Few | Low | - |  |
|  | Kercsmar 2006 | Analysis per risk factor | NA | Few | High | Performance and detection (blinding) |  |
|  | Madge 1997 | Cox regression | NA | Few | Unclear | Performance and detection |  |
| Cohort | Bloomberg 2003 | Cox regression | Adequate | NA | Low | - |  |
|  | Brittan 2017 | Log regression | Unclear | NA | Low | - | 7 |
|  | Camargo 2007 | Cox and log regression | Partial | NA | Unclear | Reporting | 7 |
|  | Chabra 1998 | Log regression | Adequate | NA | Low | - | 7 |
|  | Chen E. 2003 | Log regression | Adequate | Statistical correction | Unclear | Response | 7-8 |
|  | Chen Y. 2003 | Cox regression | Partial | Few | Low | - | 7-8 |
|  | Cinicinnati I cohort | Log and Cox regression | Adequate | NA/Few | Low | - | 8-9 |
|  | GCARS | Log and Cox regression | Adequate | NA/Few | Low | - | 9 |
|  | Giarola 2014 | Analysis per risk factor | NA | Few | Low | - | 7 |
|  | Gurk 2000 | Log regression | Unclear | NA | Unclear | Reporting | 6 |
|  | Kenyon 2014 | Log regression | Adequate | NA | Low | - | 8 |
|  | Kenyon 2015 | Log and Cox regression | Unclear | Few | Low | - | 7 |
|  | Kocev 2005 | Cox regression | Adequate | Few | Low | - | 7 |
|  | Lasmar 2006 | Log regression + survival analysis | Adequate | NA | High | Selection | 8-9 |
|  | Li 2012 | Cox regression | Adequate | NA | Unclear | Selection | 9 |
|  | Liu 2009 | Cox regression | Adequate | NA | Low | - | 9 |
|  | Minkovitz 1999 | Analysis per risk factor | NA | NA | High | Reporting | 7 |
|  | Mitchel 1994 | Cox regression | Adequate | NA | High | Reporting | 9 |
|  | Morse 2011 | Generalized linear equations | Adequate | Statistical correction | Low | - | 7 |
|  | Rasmusssen 2002 | Log regression | NA | NA | Unclear | Reporting and follow-up | 7 |
|  | Rodriguez-Mart 2014 | Poisson regression | Adequate | NA | High | Selection | 7 |
|  | Rusworth 1995 | Log regression | Partial | NA | Low | Reporting | 7 |
|  | Smiley2016 | Cox regression | Adequate | Few | Low | - | 9 |
|  | Sporik 1993 | Analysis per risk factor | NA | Few | Low | - | 7 |
|  | Taylor 1999 | Analysis per risk factor | NA | Few | Low | - | 7 |
|  | Tolomeo 2009 | Log regression | Adequate | NA | High | Selection and reporting | 8 |
|  | Walla 2004 | None | NA | NA | Low | - | 6-7 |
|  | Wu 2016 | Multivar. Poisson regression | Partial | Few | Low | - | 7 |
|  | Zipkin 2016 | Log regression | Adequate | NA | Low | - | 8 |
| Other | Bergert 2014 | Analysis per risk factor | NA | Few analysis | Low | - | 6-7 |
|  | Davis 2011 | Cox regression | Adequate | NA | High + unclear | Selection (high), follow-up and response | 7 |
|  | Fassl 2012 | Log regression | Adequate | NA | Low | - | 9 |
|  | Vicendese 2015 | Log regression | Adequate | NA | Unclear | Selection | 7 |

a: Adjustment for the most important sociodemographic factors: age, sex, ethnicity and sociodemographic status.

Abbreviations: GCARS: Greater Cincinnati Asthma Risk Study NA: Not addressed, N-O: Newcastle Ottawa Score

**E-Table 3: Association between age and risk of ED or hospital readmissions**

| Study Design | Study | Exposure | | | | | Outcome | Association | Measure | C.I. 95% | P value |
| --- | --- | --- | --- | --- | --- | --- | --- | --- | --- | --- | --- |
| Cohort | Brittan 2017 | **2-4 years** | | | | | **Hospital readmission** | **N (%)** | **92 (35.3)** |  | **0.05** |
|  |  |  |  |  |  |  | **No hospital readmission** | **N (%)** | **3519 (39)** |  |  |
|  |  | **5-11 years** | | | | | **Hospital readmission** | **N (%)** | **119 (45.6)** |  |  |
|  |  |  |  |  |  |  | **No hospital readmission** | **N (%)** | **4306 (48)** |  |  |
|  |  | **12-18 years** | | | | | **Hospital readmission** | **N (%)** | **48 (18.5)** |  |  |
|  |  |  |  |  |  |  | **No hospital readmission** | **N (%)** | **1204 (13)** |  |  |
|  | Camar 2007 | **Older age** | | | | | **ED/Hospital readmission** | **HR** | **0.98** | **0.95-0.99** | **0.032** |
|  | Chabra 1998 | 6-12 years vs 1-6y | | | | | Multiple hospitalizations | OR | 0.91 | 0.75-1.09 |  |
|  | Chen Y 2003 | **Males**  **(vs 15-19 years)** | | | **<1** | | Hospital readmission | HR | **2.36** | **2.15-2,59** |  |
|  |  |  |  |  | **1-4** | |  |  | **1.54** | **1.41-1.61** |  |
|  |  |  |  |  | 5-9 | |  |  | 1.08 | 0.98-1.19 |  |
|  |  |  |  |  | 10-14 | |  |  | 1.05 | 0.95-1.17 |  |
|  |  | **Females (vs 15-19 years)** | | | **<1** | |  |  | **1.54** | **1.42-1.66** |  |
|  |  |  |  |  | **1-4** | |  |  | **1.15** | **1.08-1.22** |  |
|  |  |  |  |  | 5-9 | |  |  | 0.87 | 0.80-0.93 |  |
|  |  |  |  |  | **10-14** | |  |  | **1.15** | **1.06-1.24** |  |
|  | Cincinn. I^a^ | Age (years, continuous) | | | | | Hospital readmission | aHR | 0.99 |  | NS |
|  | Gurk 2000 | **Age ≤5 years (vs 5-15 y)** | | | | | **Multiple hosp admissions** | **OR** | **5.12** | **2.02-12.95** | **0.02** |
|  | Kenyon 2014 | **Age (vs. 2-4 years)** | | | 5-11 y | | Hospital readmission 30d | **aOR** | 1.0 | 0.9-1.2 |  |
|  |  |  |  |  |  |  | Hospital readmission 1 y |  | 1.0 | 0.9-1.0 |  |
|  |  |  |  |  | **12-18 y** | | **Hospital readmission 30d** |  | **2.0** | **1-7-2.5** |  |
|  |  |  |  |  |  |  | **Hospital readmission 1 y** |  | **1.1** | **1.1-1.2** |  |
|  | Kenyon 2015 | **2-4 years** | | | | | **Hosp readmis (15-90d)** | **N (%)** | **681 (44)** |  | **0.001** |
|  |  |  |  |  |  |  | **No hosp read (15-90d)** | **N (%)** | **12410 (41)** |  |  |
|  |  | **5-11 years** | | | | | **Hosp readmis (15-90d)** | **N (%)** | **613 (39)** |  |  |
|  |  |  |  |  |  |  | **No hosp read (15-90d)** | **N (%)** | **13116 (44)** |  |  |
|  |  | **12-18 years** | | | | | **Hosp readmis (15-90d)** | **N (%)** | **273 (17)** |  |  |
|  |  |  |  |  |  |  | **No hosp read (15-90d)** | **N (%)** | **4565 (15)** |  |  |
|  | Li 2012 | **Age**  **(vs. 2-5 years)** | | **6-9 y** | | | **ED re-visits** | **HR** | **0.83** | **0.78-0.89** |  |
|  |  |  |  | **10-13 y** | | |  |  | **0.76** | **0.71-0.82** |  |
|  |  |  |  | **14-17 y** | | |  |  | **0.80** | **0.74-0.87** |  |
|  |  | **Age**  **(vs. 2-5 years)** | | **6-9 y** | | | **Hospital admission** | **HR** | **0.56** | **0.46-0.67** |  |
|  |  |  |  | **10-13 y** | | |  |  | **0.51** | **0.41-0.65** |  |
|  |  |  |  | **14-17 y** | | |  |  | **0.42** | **0.32-0.57** |  |
|  | Liu 2009 | **Age**  **(vs 0-4 y)** | | 5-10 y | | | Hospital readmission | HR | 0.58 | 0.64-2.03 |  |
|  |  |  |  | **11-18 y** | | |  |  | **0.57** | **0.40-0.81** |  |
|  | Mitch. 1994 | **Age <5 y (vs. 5-14 y)** | | | | | **Hospital readmission** | **aOR** | **1.71** | **1.41-2.08** |  |
|  | Rod-Mar 2014 | Age (continuous) | | | | | Hospital readmission | IRR | 1.08 | 0.39-3.03 | 0.87 |
|  | Smiley 2016 | **Age**  **(vs 12-17 y)** | | | **2-4 y** | | **ED re-visit** | **aHR** | **1.69** | **1.48-1.93** | **<**  **0.001** |
|  |  |  |  |  | **5-11 y** | |  |  | **1.24** | **1.10-1.39** |  |
|  | Wallace 2004 | Female (vs 10-14 y) | White | | | 1-4 y | Hospital readmission within 180 days of index admission | RR | 1.1 |  |  |
|  |  |  |  |  |  | 5-9 y |  |  | 0.8 |  |  |
|  |  |  | Black | | | 1-4 y |  |  | 0.6 |  |  |
|  |  |  |  |  |  | 5-9 y |  |  | 0.7 |  |  |
|  |  |  | His-panic | | | 1-4 y |  |  | 0.8 |  |  |
|  |  |  |  |  |  | 5-9 y |  |  | 1.0 |  |  |
|  |  | Male  (vs 10-14 y) | White | | | 1-4 y |  |  | 0.9 |  |  |
|  |  |  |  |  |  | 5-9 y |  |  | 0.7 |  |  |
|  |  |  | Black | | | 1-4 y |  |  | 0.8 |  |  |
|  |  |  |  |  |  | 5-9 y |  |  | 0.8 |  |  |
|  |  |  | His-panic | | | 1-4 y |  |  | 1.0 |  |  |
|  |  |  |  |  |  | 5-9 y |  |  | 1.0 |  |  |
|  | Zipkin 2016 | **Age (vs 13-17 years)** | | | **2-5 years** | | **Hospital readmission** | aOR | **0.45** | **0.25-0.80** | **0.007** |
|  |  |  |  |  |  |  | **ED re-utilization** |  | **2.23** | **1.32-3.79** | **0.003** |
|  |  |  |  |  | **6-12 years** | | **Hospital readmission** |  | **0.41** | **0.22-0.75** | **0.004** |
|  |  |  |  |  |  |  | ED re-utilization |  | 1.29 | 0.75-2.21 | 0.36 |
| Other | Vicendese 2016 | 2-6 years | | | | | ≥2 Hospital admissions | N (%) | 17 (77) |  | 0.73 |
|  |  |  |  |  |  |  | 1 hospital admission | N (%) | 16 (73) |  |  |
|  |  | 7-14 years | | | | | ≥2 Hospital admissions | N (%) | 5 (23) |  | 0.73 |
|  |  |  |  |  |  |  | 1 hospital admission | N (%) | 6 (27) |  |  |

a^:^ Auger 2015. Abbreviations: aHR: Adjusted Hazard Ratio; aOR: Adjusted Odds Ratio; CI: Confidence Interval; ED: Emergency Department; HR: Hazard Ratio; IRR: Incidence Rate Ratio; NS: Non statistically significant; OR: Odds Ratio; SD: Standard Deviation; RR: Risk Ratio.

**E-Table 4: Association between asthma severity, control and baseline treatment and risk of ED or hospital readmissions**

| Study Design | Study | Exposure | | Outcome | Association | Measure | C.I. 95% | P value |
| --- | --- | --- | --- | --- | --- | --- | --- | --- |
| Cohort | Camargo 2007 | **Pre-index OCS use** | | **ED/Hospital readmission** | **HR** | **1.10** | **1.07-1.14** | **<0.001** |
|  |  | **Pre-index SABA use** | |  |  | **1.04** | **1.02-1.05** |  |
|  | Cincinnati cohort | **Severity (ICS use in the past)** | | **Hospital readmission** | **aHR^a^** | **1.49** | **1.03-2.15** |  |
|  |  | Severity (ICS use in the past)^b^ | | Hospital readmission | aHR^c^ | 0.81 |  | NS |
|  | GCARS | Severity^d^ (vs mild intermit.) | Severe persistent | Hospital readmission | HR | 1.83 | 1.07-3.14 |  |
|  |  |  | Moderate persistent | Hospital readmission | HR | 1.70 | 1.18-2.46 |  |
|  |  |  | Mild persistent | Hospital readmission | HR | 1.12 | 0.80-1.56 |  |
|  |  | **Use of contro-llers^e^** | **Yes** | **Hospital readmission** | **N(%)** | **57 (23)** |  | **<0.001** |
|  |  |  |  | **No hospital readmission** | **N(%)** | **192 (77)** |  |  |
|  |  |  | **No** | **Hospital readmission** | **N(%)** | **45 (12)** |  |  |
|  |  |  |  | **No hospital readmission** | **N(%)** | **324 (88)** |  |  |
|  | Gurkan 2000 | **Using ICS** | | **Multiple hospital admissions** | **OR** | **0.37** | **0.16-0.86** | **0.03** |
|  | Kenyon 2015 | **≥1 controller fill** | | **Hospital readmission** | **N(%)** | **1013 (65)** |  | **<0.001** |
|  |  |  |  | **No hospital readmission** | **N(%)** | **14488 (48)** |  |  |
|  |  | **Persistent asthma** | | **Hospital readmission** | **N(%)** | **1093 (70)** |  | **<0.001** |
|  |  |  |  | **No hospital readmission** | **N(%)** | **15070 (50)** |  |  |
|  | Lasmar 2006 | **Moderate persistent vs. mild intermittent** | | **Single vs multiple hospital admissions** | **aOR** | **6.23** | **2.82-13.76** | **<0.001** |
|  |  | **No. attacks >4/month** | |  | **OR** | **2.19** | **1.03-4.71** | **0.03** |
|  | Minkovitz 1999 | **ICS as usual medication** | | **Single hospital admission** | **N(%)** | **4 (5)** |  | **0.03** |
|  |  |  |  | **Multiple hosp. admission** |  | **6 (17)** |  |  |
|  |  | Prescribed routine asthma medications | | Single hospital admission | N(%) | 65 (77) |  | 0.07 |
|  |  |  |  | Multiple hosp. admission |  | 32 (91) |  |  |
|  | Rasmussen 2002 | **FEV_1_ % predicted at 9 y** | | Single hospital admission | **Mean ±SD** | **94 ± 13** |  | **0.04** |
|  |  |  |  | Multiple hosp. admission |  | **86 ± 11** |  |  |
|  |  | FVC % predicted at 9 y | | Single hospital admission |  | 99 ± 10 |  | 0.93 |
|  |  |  |  | Multiple hosp. admission |  | 99 ± 12 |  |  |
|  |  | **FEV_1_/VC at age 9 y (%)** | | Single hospital admission |  | **84 ± 6** |  | **0.03** |
|  |  |  |  | Multiple hosp. admission |  | **78 ± 7** |  |  |
|  |  | **AHR at age 9 y** | | Single hospital admission | **N(%)** | **51** |  | **0.03** |
|  |  |  |  | Multiple hosp. admission |  | **82** |  |  |
|  | Smiley 2016 | Controller medic. (vs LTRA) | None | ED re-visit | aHR | 0.90 | 0.73-1.10 |  |
|  |  |  | ICS |  |  | 1.10 | 0.94-1.28 |  |
|  |  |  | ICS/LABA |  |  | 1.20 | 1.02-1.41 |  |
|  |  |  | ICS+LTRA |  |  | 1.19 | 0.94-1.49 |  |
|  |  |  | ICS/LABA +LTRA |  |  | 1.43 | 1.16-1.75 |  |
|  |  | **AMR ≥0.5 vs <0.5** | | **ED re-visit** | **aHR** | **0.68** | **0.58-0.81** |  |
|  | Zipkin 2016 | Discharged with ICS | | Hospital readmission | OR | 0.67 | 0.44-1.02 |  |
|  |  |  |  | Ed utilization | OR | 0.92 | 0.70-1.20 |  |

a: Adjusted for: age, etchnicity, insurance, maternal education and income; b: Auger 2015; c: adjusted for environmental exposures, medical home access, financial strain, and socioeconomic and demographic characteristics; d: Beck 2014; e: Howrylak 2014.

Abbreviations: AHR: Airway hyperresponsiveness; aHR: Adjusted Hazard Ratio; AMR: Asthma Medication Ratio; aOR: Adjusted Odds Ratio; ED: Emergency Department; FEV_1_: Forced Expiratory Volume in the first second; FVC: Forced Vital Capacity; GCASR: Greater Cincinnati Asthma Rik Study; HR: Hazard Ratio; ICS: inhaled corticosteroids; OCS: Oral corticosteroids; OR: Odds Ratio; PEFR: Peak Expiratory Flow Rate; SABA: short-acting β-adrenergic agonist; SD: Standard Deviation.

**E-Table 5: Association between asthma follow-up and management after index admission or ED visit for asthma and risk of ED or hospital readmissions**

| Study Design | Study | Exposure | | | | Outcome | | Association | Measure | C.I. 95% | P value |
| --- | --- | --- | --- | --- | --- | --- | --- | --- | --- | --- | --- |
| RCT | Gorelick 2006 | Usual Care | | | | ED re-visit (self reported) | | % | 38.4 |  | 0.90 |
|  |  | Intensive primary care linkage | | | |  |  | % | 39.2 |  |  |
|  |  | Case management programme | | | |  |  | % | 35.8 |  |  |
| Cohort | Brittan 2017 | Postdischarge outpatient visit | | | | Hospital readmission (15-90 d postdicharge) | | OR | 1.00 |  | NS |
|  | Cincinnati cohort**^a^** | **Med. home access (vs adequate)** | Almost always adequate | | | Hospital readmission | | aHR | 1.11 | 0.77-1.60 |  |
|  |  |  | **Sometimes/**  **often/never adequate** | | | **Hospital readmission** | | **aHR** | **1.56** | **1.06-2.32** |  |
|  | Li 2012 | Follow-up visit  (vs no) | | | | ED re-visit | | aHR | 0.98 | 0.93-1.03 |  |
|  |  |  |  |  |  | Hospital readmission | |  | 1.06 | 0.92-1.23 |  |
|  |  | Number FU visits  (vs 0) | | 1 | | ED re-visit | | aHR | 0.99 | 0.94-1.05 |  |
|  |  |  |  |  |  | Hospital readmission | |  | 1.05 | 0.89-1.23 |  |
|  |  |  |  | 2 | | ED re-visit | | aHR | 0.91 | 0.82-1.01 |  |
|  |  |  |  |  |  | Hospital readmission | |  | 1.12 | 0.94-1.56 |  |
|  |  |  |  | ≥3 | | ED re-visit | | aHR | 1.07 | 0.91-1.26 |  |
|  |  |  |  |  |  | Hospital readmission | |  | 0.78 | 0.48-1.29 |  |
|  |  | Type of physician for FU (vs none) | | General | | ED re-visit | | aHR | 0.98 | 0.93-1.04 |  |
|  |  |  |  |  |  | Hospital readmission | |  | 1.05 | 0.91-1.22 |  |
|  |  |  |  | Specialist | | ED re-visit | | aHR | 1.01 | 0.83-1.23 |  |
|  |  |  |  |  |  | Hospital readmission | |  | 1.41 | 0.88-2.26 |  |
|  |  |  |  | General + special. | | ED re-visit | | aHR | 0.93 | 0.71-1.21 |  |
|  |  |  |  |  |  | Hospital readmission | |  | 0.86 | 0.38-1.93 |  |
|  | Minkovitz 1999 | **Post-discharge visit** | | | Peadiatrician | Single hospital admission | | N(%) | 77 (92) |  | 0.28 |
|  |  |  |  |  |  | Multiple hosp. admission | |  | 34 (97) |  |  |
|  |  |  |  |  | Allergy | Single hospital admission | | N(%) | 10 (12) |  | 0.40 |
|  |  |  |  |  |  | Multiple hosp. admission | |  | 6 (18) |  |  |
|  |  |  |  |  | **Pulmonary** | **Single hospital admission** | | **N(%)** | **10 (12)** |  | **0.002** |
|  |  |  |  |  |  | **Multiple hosp. admission** | |  | **13 (37)** |  |  |
|  | Morse 2011 | CAC-3 compliance  (5% improvement) | | | | ED re-visit | 30 Days | OR | 0.97 | 0.90-1.04 | 0.36 |
|  |  |  |  |  |  |  | 90 Days |  | 0.96 | 0.77-1.18 | 0.68 |
|  |  |  |  |  |  | Hospital readmission | 30 Days |  | 0.99 | 0.96-1.02 | 0.53 |
|  |  |  |  |  |  |  | 90 Days |  | 1.01 | 0-90-1.12 | 0.90 |
|  |  |  |  |  |  | Multiple hosp. admission | |  | **82** |  |  |
|  | Smiley 2016 | **Follow-up appointment (vs no)** | | | | **ED re-visit** | | **aHR** | **0.86** | **0.76-0.93** | **0.002** |
|  | Zipkin 2016 | **HMPC compliance (partial/full vs none)** | | | | **Hospital readmission** | | **OR** | **0.63** | **0.41.0.95** | **0.028** |
|  |  |  |  |  |  | **Ed re-utilization** | | **OR** | **0.73** | **0.56-0.96** | **0.022** |
| Other | Bergert 2014 | **Post-CAC Implementation vs Pre-CAC** | | | | ED re-visit | 0-30 days | **OR** | N/A | N/A |  |
|  |  |  |  |  |  |  | 31-90 days |  | 1.60 | 0.44-5.81 |  |
|  |  |  |  |  |  |  | 91-180 d |  | 0.57 | 0.20-1.67 |  |
|  |  |  |  |  |  | **Hospital readm.** | 0-30 days |  | 0.29 | 0.05-1.73 |  |
|  |  |  |  |  |  |  | 31-90 days |  | 0.87 | 0.21-3.50 |  |
|  |  |  |  |  |  |  | **91-180 d** |  | **0.29** | **0.11-0.78** |  |
|  | Fassl 2012 | **Post-CAC Implementation vs Pre-CAC** | | | | **Hospital readmission** | | **aOR** | **0.67** | **0.50-0.91** | **0.01** |

a: Auger 2013. Abbreviations: RCT: Randomized Clinical Trial; HR: Hazard Ratio; aHR: Adjusted Hazard Ratio, OR: Odds Ratio; aOR: Adjusted Odds Ratio; ED: Emergency Department; FU: follow-up; CAC: Childrens’ Asthma Care; HMPC: Home Management Plan Care (part of the CAC); N/A: not applicable.
